# Trophic transfer of CeO_2_ nanoparticles from clamworm to juvenile turbot and related changes in fish flesh quality

**DOI:** 10.1016/j.eehl.2025.100174

**Published:** 2025-08-05

**Authors:** Liyun Yin, Zhuomiao Liu, Jian Zhao, Shu Chen, Xiaochuan Wang, Zhenyu Wang

**Affiliations:** a College of Life Sciences, Hebei Key Laboratory of Animal Physiology, Biochemistry and Molecular Biology, Hebei Collaborative Innovation Center for Eco-Environment, Hebei Normal University, Shijiazhuang 050024, China; b Institute of Coastal Environmental Pollution Control, Laboratory of Marine Environment, and Ecology and Frontiers Science Center for Deep Ocean Multispheres and Earth System, Ocean University of China, Qingdao 266100, China; c Institute of Environmental Processes and Pollution Control, and School of Environment and Ecology, Jiangnan University, Wuxi 214122, China

**Keywords:** Engineered nanoparticles, CeO_2_, Trophic transfer, Flesh quality, Food safety

## Abstract

Engineered nanoparticles (ENPs) accumulate in marine sediments and exhibit adverse effects on benthic organisms. However, the effect of ENPs on marine benthic food chains is largely unknown. Herein, we investigated the trophic transfer and transformation of CeO_2_ ENPs within a simulated marine benthic food chain from clamworm (*Perinereis aibuhitensis*) to turbot (*Scophthalmus maximus*), as well as their effects on fish flesh quality. The results showed that Ce contents in turbot increased with the accumulation of CeO_2_ ENPs in clamworm, but no biomagnification of CeO_2_ ENPs occurred along this food chain. During trophic transfer, CeO_2_ ENPs in turbot experienced transformation from Ce(IV) to Ce(III). Importantly, CeO_2_ ENPs accumulated in the muscle of turbot and decreased the crude protein, total amino acid, and delicious amino acid contents, as well as the texture of the muscle. CeO_2_ ENPs induced the deterioration of flesh quality, which was mainly related to metabolism in muscle and intestinal disorders caused by oxidative stress. Specifically, CeO_2_ ENPs increased the relative abundance of *Stenotrophomonas* and *Vibrio* in the turbot intestine*,* while decreasing those of *Lactobacillus*, *Bacillus*, and *Acinetobacter*. Significant disturbances in purine and amino acid (aspartate, glutamate, glycine, etc.) metabolism in muscle were induced by CeO_2_ ENPs. Moreover, correlation analysis showed that microbiota dysbiosis was highly correlated with muscle metabolic dysfunction. Our study provides insights into the transfer and transformation of CeO_2_ ENPs and their interference with fish flesh quality via the gut–muscle axis, providing useful information on assessing ecological risk and food safety in marine environments.

## Highlights

• Biomagnification of CeO_2_ ENPs did not occur along clamworm-turbot transfer.
• Biotransformation of CeO_2_ ENPs to Ce(III) was found in turbot after trophic transfer.
• CeO_2_ ENPs reduced flesh security, protein quality, umami and taste in turbot.
• The flesh texture and flavor deterioration might be caused by the metabolism in muscle and intestinal disorder.

## Graphical abstract

**Figure:**
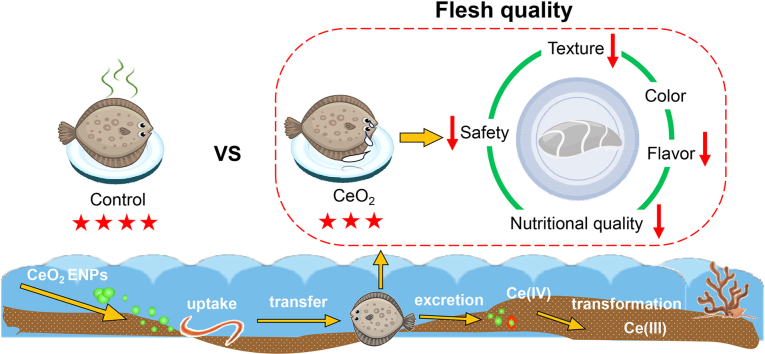

## 1 Introduction

As contaminants of emerging concern, engineered nanoparticles (ENPs) are distributed in soil and natural waters, and environmental concentrations of ENPs are dramatically increasing with the widespread application of nanoproducts [1]. As one of the main sinks, marine benthic environments have much higher concentrations of ENPs than other compartments, thereby affecting marine benthic organisms [2,3]. Therefore, the behavior and ecological effects of ENPs in the marine benthic environment deserve attention.

The fate and transport of ENPs are affected by marine benthic organisms through bioturbation, accumulation, and biotransformation [2,4,5]. Notably, these aquatic species are tied together through the food web in nature. This predation relationship may transfer ENPs to higher-level consumers while causing toxic hazards [[6], [7], [8], [9]]. Specifically, marine medaka (*Oryzias melastigma*), after being fed with Ag ENPs-contaminated brine shrimp (*Artemia salina*), exhibited reduced water content and growth [7]. Turbot (*Scophthalmus maximus*), after being fed with TiO_2_ ENPs-treated clamworm (*Perinereis aibuhitensis*), altered the nutritional composition of fish [2]. Trophic transfer of amine-functionalized polystyrene nanoparticles through a microalga–crustacean–small yellow croaker food chain inhibited digestive enzyme activity in fish [8]. Particularly, Cu ENPs accumulated in the algae (*Pseudokirchneriella subcapitata*) can be biomagnified in the mysid (*Limnomysis benedeni*) [9]. In this case, small changes in the high-trophic-level predators may cause instability in food webs, leading to a potential influence on entire ecosystems. However, most studies use single organisms to assess the toxic effects of ENPs in the marine environment, ignoring the trophic transfer of ENPs along the food chain, which may cause more serious harm to high-trophic-level organisms [2,7,8]. Moreover, very little is known about the effects of ENPs on the marine benthic food chain, and a better understanding of their ecotoxicological risk is certainly warranted.

In addition to physiological toxicity, increasing concern has been raised regarding the nutritional quality of organisms after ENP exposure [[10], [11], [12]]. Specifically, ENPs can not only accumulate in aquatic organisms such as marine fish, crayfish, and whiteleg shrimp, but also reduce their growth and nutritional quality (condition factor, microelements, crude protein, and amino acid contents, etc.) [2,[10], [11], [12]]. Among them, as the main body of marine biological resources, fish are high-trophic-level organisms that provide approximately 16% of the world's high-quality animal protein intake and yield significant economic benefits [13]. Muscle is the primary edible tissue of fish for consumers, and muscle mass directly affects the quality of fish products and human health [14]. Then, it is particularly important to explore the effect of ENPs on fish flesh quality. Notably, flesh quality is a multifaceted trait, encompassing nutritional quality, food security, and satisfaction, including color, texture, and flavor [15,16]. However, so far, reports on fish after ENPs exposure are limited to nutritional quality and safety [2,4,10], and little concern has been given to the effects on muscle satisfaction [15]. Importantly, texture-related satisfaction (tenderness, flesh color, flavor, etc.) is the primary criterion for consumers to assess flesh quality initially, which is shaped by muscle development and growth [15,16]. Nevertheless, the most targeted organs in ENPs toxicology studies are the intestine and liver to explain the effects on nutrient quality [2,4,17], whereas the effects on muscle tissue are rarely studied [11]. In fish, the color, flavor, and texture of flesh quality are related to the structural integrity of muscle tissue, water-holding capacity, collagen content, lipid content, and flavor compounds in muscle tissue [[14], [15], [16]]. These attributes are closely associated with the physiology and metabolism of muscle tissue, particularly oxidative damage [12,14,16]. Most importantly, numerous studies have identified that ENPs disturb the antioxidant defense system, muscular tissue structure, intestinal structure and function, as well as metabolism in fish [10,11,18], which are related to flesh quality [2,11,12]. Thus, ENPs are likely to be transferred to fish via the marine benthic food chain and interfere with the growth and flesh quality of fish.

CeO_2_ ENPs, among the top 10 most produced ENPs globally, are widely used in agriculture and fuel additives [19], and their annual global production is estimated to reach 10,000 ​t by 2050 [20]. The reported environmental concentration of CeO_2_ ENPs is 40 ​μg/L in surface water [21] and at “mg/L” in hotspot environments, such as wastewater treatment plant effluent and biosolids [22]. Due to their poor suspension stability in aquatic systems, CeO_2_ ENPs tend to settle at the bottom [23]. By 2050, the release of CeO_2_ ENPs to aquatic sediments could reach up to 13.5 ​mg/kg [20]. It is noted that Ce has been detected in naturally occurring marine benthic fish [megrim, 6.6 ​ng/g DW (dry weight)] and razor shell (454 ​ng/g DW) [24]. Recent studies indicated that CeO_2_ ENPs at 5–100 ​mg/L could accumulate in organisms, exerting adverse impacts on their growth and nutritional composition [6,10,25,26]. Particularly, biomagnification was observed in a radish-snail food chain after exposure to 100 ​mg/L CeO_2_ ENPs [6]. Thus, CeO_2_ ENPs were chosen as the model ENPs for this study. Subsequently, clamworm and turbot were selected as the tested creatures. Clamworm, a benthic invertebrate, serves as the main food source for fish and plays a key role in marine benthic ecosystems by transferring pollutants to higher-level consumers [2]. Turbot, a high-trophic-level benthic carnivorous fish, is one of the most commercially valuable aquaculture species in Europe and Asia [2]. Thus, the aims of this study are to (1) explore the transfer, transformation, and accumulation of CeO_2_ ENPs from clamworm to turbot; (2) comprehensively evaluate the effect of CeO_2_ ENPs on the growth and flesh quality of turbot (e.g., nutritional quality, food security, color, texture, and flavor); (3) elucidate the mechanism of changes in flesh quality in turbot after CeO_2_ ENPs exposure. The results will provide new insights into better understanding marine ecosystem risk and food safety.

## 2 Materials and methods

### 2.1 CeO_2_ ENPs characterization

Size, morphology, and the surface area of CeO_2_ ENPs (Sigma-Aldrich, USA) were characterized with transmission electron microscopy (TEM, H-7650, Hitachi, Japan) and adsorption–desorption of N_2_ using Autosorb-1 (Quantachrome, USA), respectively. CeO_2_ ENPs, after preparation in seawater and deionized water, were subjected to bath sonication for 30 ​min. Then, the zeta potential and hydrodynamic diameter of CeO_2_ ENPs were measured by Zetasizer Nano (Nano-ZS90, Malvern, UK). To assess cerium ion release, the seawater-dispersed CeO_2_ suspension (100 ​mg/L) was oscillated for 24 ​h at 18 ​°C. Subsequently, the dissolved cerium ion content was quantified by inductively coupled plasma mass spectrometry (ICP-MS, PerkinElmer NexION 350X, USA).

### 2.2 Waterborne exposure of CeO_2_ ENPs to clamworms

The purchased clamworms (2.79 ​± ​0.29 ​g) from Qingdao Honzenda Company (China) were acclimated in seawater for 3 days and then fasted for 3 days before exposure (temperature 18 ​± ​1 ​°C, salinity 30‰ ​± ​1.0‰). Every 10 healthy clamworms were stocked in seawater in a culture tank (1 ​L) with CeO_2_ ENPs for 24 ​h at 0, 10, 50, and 100 ​mg/L, respectively. According to an 88.7% sedimentation rate of CeO_2_ ENPs in the aquatic food web [23], the actual concentrations were approximately 1.13, 5.65, and 11.3 ​mg/L in this water, which are highly correlated with the environment-related concentrations. Each treatment had 150 replicates. After 24 ​h of waterborne exposure, the clamworms were rinsed with deionized water four times to remove the adsorbed CeO_2_ ENPs (Table S1) and subsequently stored at −80 ​°C for the following feeding experiments. In addition, 10 clamworms were exposed to 100 ​mg/L CeO_2_ ENPs for 14 days and subsequently depurated for 1 day in clean seawater. The whole volume of cultured seawater was renewed every day. On Day 15, the clamworms were collected and washed to be stored at −80 ​°C for Ce speciation analysis.

### 2.3 Dietary exposure of CeO_2_ ENPs from clamworm to turbot

Juvenile turbots were purchased from Qinhuangdao aquatic market (China). Juvenile turbots were acclimated for 14 days and trained to eat clamworms. The water temperature, pH, and salinity were 18 ​± ​1 ​°C, 8.0 ​± ​0.1, and 25‰ ± 1.5‰, respectively. Before dietary exposure**,** all the fish were fasted for 24 ​h.

Dietary exposure included a 20-day uptake period, followed by a 7-day depuration period. 36 healthy fish (42.5 ​± ​0.4 ​g) were randomly distributed into a 300-L tank (4 replicates for each treatment). During the uptake period (Day 1–20), fish were fed with CeO_2_ ENPs-exposed clamworms at 10, 50, and 100 ​mg/L twice per day [8:00 and 17:00, 5% of the fish's WW (wet weight)], which were designated as 10DE, 50DE, and 100DE, respectively. After being exposed for 20 days, fish were fed again with uncontaminated clamworms during the depuration period (Day 21–27). For the uptake and depuration periods, every four fish were sampled on days 0, 5, 10, 15, 20, 22, 24, and 27 from each treatment for Ce determination, respectively. After the 20-day uptake period, all the fish were further fasted for 24 ​h. Six fish were randomly selected from each tank, and blood samples were drawn from their caudal veins. Subsequently, the gill, skin, muscle, brain, eye, liver, stomach, and intestine of fish were immediately excised and weighed for Ce analysis. After 27 days of exposure, the remaining fish, after fasting for 24 ​h, were weighed and measured for body length. Three fish per tank were collected to determine the fish body composition. The remaining fish were then dissected, and the liver and viscera were weighed to measure the hepatosomatic index and viscerosomatic index. The dorsal muscle from the body was sampled for flesh quality analysis, and the sampling locations are shown in Fig. S1. During the dietary exposure, feces were collected every day, and then the entire volume of cultured seawater was replaced to maintain a clean breeding environment.

### 2.4 Ce determination and Ce speciation analysis by X-ray absorption near edge structure (XANES)

To determine sample Ce content, all samples were analyzed by ICP-MS [2,10].

BCF = *C*_clamworms_/*C*_seawater_

BMF = *C*_fish_/*C*_clamworms_

where bioaccumulation factor (BCF) represents the ratio of the content of Ce in clamworms to that in seawater (*C*_seawater_, μg/g); biomagnification factor (BMF) represents the ratio of the content of Ce in fish to that in clamworms; *C*_clamworms_ is the content of Ce in clamworms (μg/g); *C*_fish_ is the content of Ce in fish (μg/g).

To analyze the Ce speciation, the dry clamworms, turbot tissue/organ, and feces samples were analyzed at the Shanghai Synchrotron Radiation Facility (SSRF, Shanghai, China) on the beamline BL14W1. CeO_2_ ENPs, CeAC_3_, CePO_4_, and Ce-cysteine were used as reference compounds. The Ce XANES spectra were determined by a linear combination fit (LCF) using Athena software [10].

### 2.5 Growth performance and body composition of turbots

After the 27-day dietary exposure, the growth performance of turbots was calculated as follows [27]: weight gain rate (WGR), morbidity, survival rate (SR), viscerosomatic index (VSI), condition factor (CF), hepatosomatic index (HSI), and moisture, crude protein, ash, and crude lipid content of the fish body were analyzed in Text S1.

### 2.6 Flesh quality analysis and antioxidant capacity

Muscle color indicators (a∗/redness, L∗/lightness, b∗/yellowness), the water-holding capacity (WHC), and texture profile analysis (TPA) of the dorsal muscle were determined by a Minolta Chroma Meter (CR-10, Osaka, Japan) and Universal TA texture analyzer (Tengba, China), respectively [15,16]. Therein, WHC was measured as described by Yang et al. [28], including steaming loss, drip loss, and centrifugal loss.

Amino acid composition of flesh was measured by Ultra Performance Liquid Chromatography (UPLC, Waters Acquity, USA), as described by Yang et al. [28]. The collagen content, glutathione peroxidase (GSH-Px), superoxide dismutase (SOD), total antioxidant capacity (T-AOC), lactate (LA), and malondialdehyde (MDA) levels in muscle were analyzed with assay kits (Nanjing Jiancheng, China) following the supplier's detailed protocol.

### 2.7 Intestine structure and microbiota analysis

The fresh intestine samples of turbot on day 27 were immersed in 4% paraformaldehyde, then made into paraffin sections and stained with hematoxylin-eosin. The intestinal sections were visualized using an imaging microscope (BX51T-PHD-J11, Olympus, Japan) and Image-Pro Plus software. Contents from the distal intestinal tissue of turbot were taken on day 27, and then microbiota analysis was conducted based on Text S2.

### 2.8 Flesh metabolic analysis

The 6 dorsal muscle samples of fish (Day 27) were taken to perform untargeted metabolomics analysis. The metabolic changes in the muscles were analyzed using UPLC-QTOF-MS (1290 UHPLC-Agilent 6550/SCIEX 6600, Agilent, USA). The methods for extracting, separating, and identifying the metabolites were based on the approach of Peng et al. [14]. According to the Orthogonal Partial Least Squares Discriminant Analysis (OPLS-DA), Variable Importance in Projection (VIP) ​> ​1 and *p*-value < 0.05 were applied as screening criteria for identifying differential metabolites. The coefficient analysis and intergroup relationships were analyzed using the R package.

### 2.9 Statistical analysis

One-way ANOVA with Duncan's test was performed using STATISTICA 6.0. Results were presented in the form of the mean ​± ​standard deviation (SD). A significant level was considered as *p* ​< ​0.05.

## 3 Results and discussion

### 3.1 Transfer of CeO_2_ ENPs from clamworm to turbots

According to TEM imaging (Fig. S2A), the individual size of CeO_2_ ENPs was around 20 ​nm with an irregular shape. As shown in Table S2, the surface area of CeO_2_ ENPs was 41.5 ​m^2^/g. The zeta potential of CeO_2_ ENPs was 23.2 ​mV in deionized (DI) water. The hydrodynamic diameter of CeO_2_ ENPs in seawater (1647 ​nm) was greater than in DI water (131.8 ​nm), showing severe agglomeration of CeO_2_ ENPs in seawater. This was confirmed by our observation in Fig. S2B. Moreover, the released ionic Ce was not detected in seawater after 24 ​h of incubation.

**Fig. 1:**
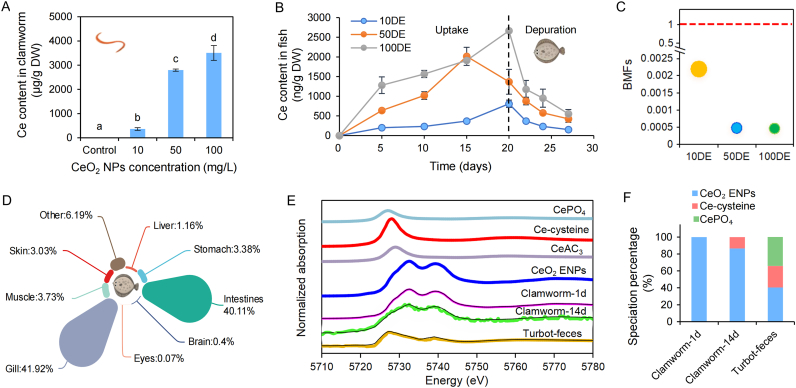
Transfer and transformation of CeO_2_ ENPs from clamworm to turbot. (A) Ce contents in clamworm after being exposed to 0–100 ​mg/L of CeO_2_ ENPs for 24 ​h; (B) Ce contents in turbot after dietary exposures, Day 0–20 and Day 21–27 were the uptake period and depuration period, respectively; (C) Biomagnification Factors (BMFs) of Ce in juvenile turbots; (D) Distribution of CeO_2_ ENPs in turbot; (E) XANES spectra and (F) the percentage of Ce species in clamworm and turbot feces, respectively. In panel (E), the dotted line indicates the fitting line. Different letters indicate significant difference (*n* ​= ​3, *p* ​< ​0.05).

As described in Fig. 1A, Ce contents in clamworms gradually increased with increasing exposure concentrations of CeO_2_ ENPs (10–100 ​mg/L) after waterborne exposure for 24 ​h, indicating that Ce accumulation in clamworms exhibited a concentration-dependent relationship with CeO_2_ ENPs. Although most of CeO_2_ ENPs were excreted in feces (Fig. S3), the high accumulation (>300 ​μg/g DW) (Fig. 1A) still existed and possibly transferred to higher-level consumers. The BCFs of Ce in clamworms were 36.2, 57, and 34.3 after exposure to CeO_2_ ENPs at concentrations of 10, 50, 100 ​mg/L, respectively (Table S3). Despite the high accumulation of Ce in clamworms, the 24-h survival of clamworms across all exposure groups remained at 100%, and their nutritional parameters showed no significant changes (Table S4).

After being fed with CeO_2_ ENPs-treated clamworms, Ce content in turbots exhibited a time- and concentration-dependent increase during the uptake period (Days 0–20) (Fig. 1B), indicating that the body burden of CeO_2_ ENPs in predatory turbot exhibited a positive correlation with CeO_2_ content in their prey, clamworms. Subsequently, Ce contents rapidly decreased during a 7-day depuration (Days 21–27), but 17.7%–31.1% of Ce contents still remained in fish body at Day 27. Nevertheless, the BMFs of turbots were much less than 1 ​at 10, 50 and 100 ​mg/L CeO_2_ ENPs (Fig. 1C), showing no biomagnification of CeO_2_ ENPs from clamworms to turbots. We found that the amount of Ce content in feces was much higher (1241–12,932 ​μg/g) than that in all the tissues/organs (Fig. S4), suggesting that the rapid elimination of CeO_2_ ENPs through feces should be a key factor for the insignificant biomagnification. Importantly, these ENPs will re-enter aquatic environments via fecal excretion and once again become food sources for other benthic omnivorous organisms (e.g., crab, clamworm), posing a more complex ecological risk [2,10]. Moreover, the feces of turbots readily disintegrated under aeration, enabling CeO_2_ ENPs in the feces to be bioavailable for turbots again. Regarding the distribution of Ce in fish, it was primarily found in the gill, followed by the intestine, stomach, muscle, skin, liver, brain, and others (Fig. 1D). Tissue accumulation of CeO_2_ ENPs in predator fish was positively related to their content in prey clamworms (Fig. S4A–F). Notably, the detected Ce contents in the blood of turbots showed that CeO_2_ ENPs may be absorbed by capillaries in gills or digestive tract, then migrate into the bloodstream, and eventually enter different tissues/organs through the circulatory system (Fig. S4G). Most notably, as the main edible parts of turbot, 3.73% of Ce accumulated in muscle, which was the main enriched organ besides digestive system and gill, showing that turbot fed CeO_2_ ENPs-treated clamworms exhibited potential flesh quality problems and food safety risks to human beings. Ce was detected in the serum (0.058 ​μg/L) and urine (0.03 ​μg/L) of pregnant women [29,30], and oral ingestion (e.g., the consumption of Ce-containing seafoods) should be one of important pathways.

In Fig. 1E, Ce species in the clamworm and turbot feces were analyzed by XANES. The Ce contents of tissue/organ in turbot were too low (<5 ​ppm) to be characterized by XANES. As shown in Fig. 1F, after water exposure to clamworms, there was no transformation of CeO_2_ in clamworms at Day 1, showing that CeO_2_ kept its original form in clamworms. After transfer from clamworm to turbot, only 40.5% of CeO_2_ ENPs remained in the feces of turbot, with the other part of Ce(IV) transformed into Ce(III), including Ce-cysteine (25.3%) and CePO_4_ (34.2%). Interestingly, clamworms after exposure to CeO_2_ ENPs for 14 days exhibited a lower proportion of Ce(III) as Ce-cysteine (13.4%). Thus, the transformation of CeO_2_ ENPs was determined by exposure time and the organism species. Specifically, the different transformation efficiencies of CeO_2_ ENPs between clamworm and turbot may be attributed to their digestive systems, including the pHs of digestive fluids, digestive enzymes, and gut microbes. As for fish, the digestive tract provides a more acidic environment in the stomach (pH ​< ​5.0) [10], richer digestive enzymes, and microbes [18] than that in clamworms (no stomach), thereby enhancing the dissolution and transformation efficiencies of CeO_2_ ENPs in turbot feces. It is observed that CeO_2_ ENPs may increase the abundance of sulfatase-producing gut microbiota and potentially induce intestinal inflammation, thereby promoting the biotransformation of Ce(IV) to Ce(III) species [10]. Moreover, CeO_2_ ENPs are tightly associated with ROS scavenging ability, impacting the antioxidant system in organisms [31]. The study found that 12.8% of CeO_2_ ENPs were transformed to Ce(III) after interaction with planarian (a benthic animal in the water body), and the biotransformation of CeO_2_ ENPs reduced antioxidant defense system, leading to delayed planarian regeneration and affecting the mobility [32]. Conversely, CeO_2_ ENPs enhanced the salt tolerance of cucumber, which is linked to their early induction of the antioxidant defense system [31]. The bio-effects of CeO_2_ ENPs remain controversial. The toxicity of CeO_2_ ENPs may depend on the species, dose, and physiochemical properties of CeO_2_ ENPs [6,10,31,32].

### 3.2 Growth performance, nutrition and flavor of fish

**Fig. 2:**
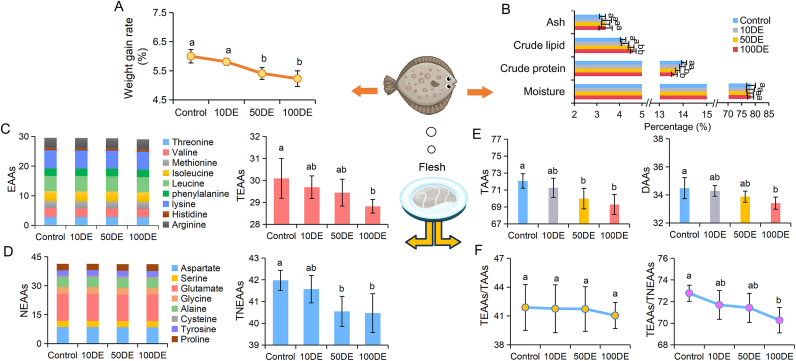
Growth performance (A), body composition (B), and flesh amino acid composition (g/100 ​g WW) (C–F) of turbot after being fed with CeO_2_ ENPs-treated clamworms for 0, 10, 50, 100 ​mg/L (designated as control, 10DE, 50DE, and 100DE). EAAs, essential amino acids; NEAAs, total non-essential amino acids; TEAAs, total essential amino acids; TNEAAs, total non-essential amino acids; TAAs, total amino acids; DAAs, delicious amino acids. Different letters indicate significant difference (*n* ​= ​3, *p* ​< ​0.05).

As shown in Fig. 2A and Table S5, the growth performance (SGR and WGR) in turbots gradually reduced as the concentrations of CeO_2_ ENPs increased, especially in 50DE and 100DE groups (*p* ​< ​0.05). Moreover, although there were no significant differences in SR, CF, and VSI in all groups (*p* ​> ​0.05), a significant increase in HSI was observed in 100DE group (*p* ​< ​0.05). Particularly, the morbidity of fish significantly increased with CeO_2_ ENPs dietary exposure as follows: 100DE ​> ​50DE ​> ​10DE ​> ​control (*p* ​< ​0.05). In Fig. 2B, compared to the control group, no significant difference in moisture and ash of body composition was observed among all the groups (Table S6), whereas lower crude protein and higher crude lipid were determined in 50DE and 100DE groups (*p* ​< ​0.05), showing a change in nutritional composition of turbots under CeO_2_ ENPs dietary exposure.

Marine fish provide high-protein food with lower fat [2]. As given in Fig. 2C–E and Table S7, the contents of total nonessential amino acids (TNEAAs), total essential amino acids (TEAAs), and total amino acids (TAAs) in flesh of turbot showed an obviously negative correlation with Ce contents in clamworms (*p* ​< ​0.05), which was consistent with the observed lower crude protein in this study. Particularly, turbots fed with CeO_2_ ENPs-exposed clamworms significantly influenced nonessential amino acids (NEAAs) such as aspartate, glutamate and glycine (*p* ​< ​0.05) (Fig. S5). Notably, aspartate, glutamate, glycine, and alanine are the delicious amino acids (DAAs), and their contents determine how delicious fish tastes [16]. In this study, the contents of DAAs in flesh of turbot gradually decreased as the contents of CeO_2_ ENPs increased in clamworms (*p* ​< ​0.05) (Fig. 2E), stating that the flesh of turbot had poor flavor. Furthermore, according to the ideal model of FAO/WHO, TEAAs/TAAs > 40% and TEAAs/TNEAAs > 60% indicate better protein quality [33]. It seems that the amino acid composition of turbot meets the requirements of the ideal model, and all remained classified as high-quality protein after CeO_2_ ENPs dietary exposure (Fig. 2F). However, although there were no effects on TEAAs/TAAs in flesh among all the groups (*p* ​> ​0.05), TEAAs/TNEAAs gradually decreased with Ce contents in clamworms, especially in 100DE group (*p* ​< ​0.05) (Fig. 2F), indicating that the protein quality of fish was gradually deteriorating after CeO_2_ ENPs dietary exposure. Moreover, alterations in amino acid composition in flesh affected its flavor [16], which is consistent with our results. Excessive lipid content can negatively impact flesh firmness and processing quality, and induce a fishy odor via oxidation during storage, leading to uneven flesh color distribution [34], ultimately reducing the consumption appeal of turbot. Thus, potential exposure to ENPs in aquaculture warrants attention, as the deterioration of nutritional quality in fish could potentially lower the sustainable development of the fishing industry.

### 3.3 Flesh color, water-holding capacity, and texture

**Fig. 3:**
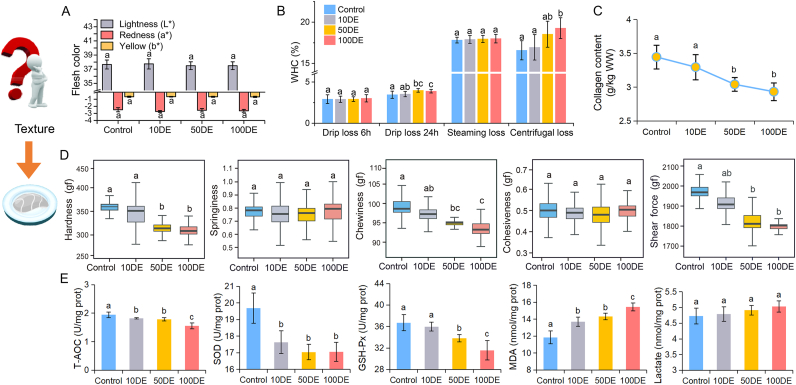
Flesh color (A), water-holding capacity (B), collagen content (C), texture profile (D), and oxidative stress (E) of turbot after being fed with CeO_2_ ENPs-treated clamworms (0, 10, 50, 100 ​mg/L). Different letters indicate significant difference (A-D, *n* ​= ​6; E, *n* ​= ​4; *p* ​< ​0.05).

As described in Fig. 3A–D, there was no significant effect on flesh color, including redness, lightness, and yellowness of turbots fed with CeO_2_ ENPs-treated clamworms (*p* ​> ​0.05). However, WHC, collagen content, and texture profile of flesh were significantly affected by CeO_2_ ENPs dietary exposure (*p* ​< ​0.05). Among these, WHC can directly affect flesh quality, including color, taste, texture, and flavor. WHC is typically evaluated by steaming loss, drip loss, and centrifugal loss [16]. The centrifugal loss is primarily attributed to the loss of free water from muscle tissue [35]. Drip loss mainly results from the loss of bound water distributed within muscle structures, which is caused by the modification, denaturation, and structural changes of proteins [36]. Thus, enhancing muscle WHC can improve muscle structural integrity, thereby directly enhancing flesh quality [16,28]. Nevertheless, higher 24-h flesh drip loss and centrifugal loss indicated a decrease in WHC in turbot muscle. Additionally, collagen, the major component of connective tissues, plays a vital role in maintaining flesh integrity. Its content is closely associated with flesh springiness and hardness [16]. In this work, decreased WHC and collagen content damaged the structural integrity of turbot muscle, potentially influencing flesh texture and flavor following exposure to CeO_2_ ENPs.

Importantly, fish flesh with high firmness and elasticity is more acceptable to consumers [15]. Improving the flesh hardness (termed firmness) can enhance the flesh quality, and lower shear force increases flesh tenderness [16]. Nevertheless, lower shear force, chewiness, and cohesiveness reduce the binding ability between fish muscle cells, making it impossible to resist damage and maintain structural integrity, leading to a bad taste with lower elasticity in muscle [15,28]. In this study, springiness and cohesiveness showed no significant difference among all groups (*p* ​> ​0.05). However, hardness, shear force, and chewiness were markedly decreased in treated fish compared to the control group (*p* ​< ​0.05), which potentially results in poor taste quality of turbot muscle after dietary exposure to CeO_2_ ENPs.

### 3.4 Antioxidant capacity of flesh

As shown in Fig. 3E, the activities of T-AOC, SOD and GSH-Px in turbot muscle significantly decreased, whereas MDA contents increased, showing that turbot fed with CeO_2_ ENPs-treated clamworms caused severe oxidative stress and lipid peroxidation in muscle, especially in 50DE and 100DE groups (*p* ​< ​0.05). Importantly, the antioxidant capacity of muscle is a critical determinant of flesh quality. Oxidative damage has been observed to reduce WHC in flesh, disrupting the structural integrity of muscle tissue [15]. This disruption triggers the release of proteases that hydrolyze muscle fibers, resulting in decreased muscle shear force, loose flesh texture, and deteriorated texture quality [14]. Beyond that, high levels of lactate can decrease muscle pH, induce protein denaturation, and reduce flesh WHC, resulting in a stiff texture with poor taste and color [28]. In this work, no significant differences were observed in lactate contents (*p* ​> ​0.05), but lactate contents had a slight increase in 50DE and 100DE groups, indicating poor flesh tenderness. As revealed in previous research, oxidative stress induces ROS generation, causing oxidative damage that destroys muscle structural integrity and alters flesh quality [12,14]. Excessive ROS production results from ENP exposure, which decreases antioxidant defenses and thereby suppresses antioxidant activity [5,10,25]. Moreover, high levels of CeO_2_ ENPs impaired the activity of antioxidant enzymes, thereby negatively affecting the nutritional composition of carp and crayfish [10]. Based on the flesh antioxidant analysis in this study, turbot fed with CeO_2_ ENPs-treated clamworms showed decreased antioxidant capacity, induced oxidative damage in muscle, thereby resulting in poorer flesh texture.

### 3.5 Intestinal structure and microbiota analysis

**Fig. 4:**
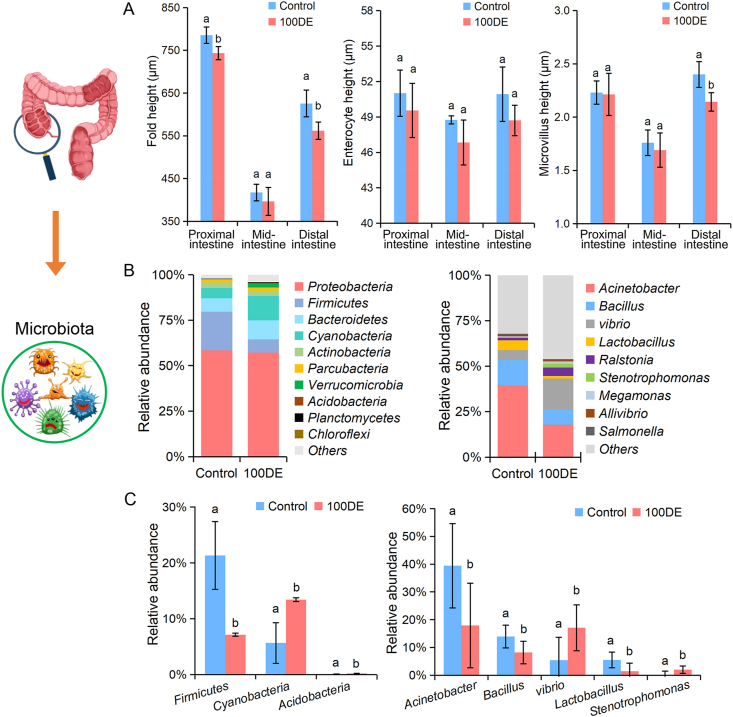
The structure and community composition of intestine in turbot after dietary exposure to CeO_2_ ENPs (100 ​mg/L, 100DE). (A) The intestine structure; (B) Taxonomy classification at the phylum level (left) and at the genus level (right) in gut microbiota (the top 10); (C) Significant variations in the relative abundance of intestinal microbiota at the phylum (left panel) and genus (right panel) levels in turbot. Different letters indicate significant difference (*n* ​= ​4, *p* ​< ​0.05).

Intestinal structure and microbiota of turbot were observed in the 100DE group to determine potential effects on growth and flesh quality. The results showed that turbot fed CeO_2_ ENPs-treated clamworms markedly caused injury to the epithelial microvilli of the intestine (Fig. S6), and a significantly lower microvillus height and fold height of distal-intestines were observed in comparison with the control (Fig. 4A), potentially decreasing intestinal digestive enzymes and nutrient utilization. The main reason was that the internalized ENPs caused physical damage to the intestinal histological structures [2], which was confirmed by high accumulation content in the intestine and the sharp, irregularly shaped CeO_2_ ENPs. Importantly, gut digestive enzymes and nutrient utilization are typically associated with the intestinal microbiota, which are positively linked to growth performance [[17], [28]]. Generally, microbial community is comprehensively evaluated by Chao1, Shannon, and Simpson indices, etc [18]. The changes in these indices collectively indicated that the alpha diversity of turbot intestinal microbiota was significantly increased after CeO_2_ ENPs dietary exposure (Fig. S7).

At the phylum level, the abundances of *Cyanobacteria* and *Acidobacteria* increased significantly, whereas *Firmicutes* decreased markedly in 100DE group compared to the control (Fig. 4B). Notably, most *Cyanobacteria* are producers of cyclic lipopeptides (inducing cell necrosis) and toxic microcystin, which can induce oxidative stress in aquatic organisms [37,38]. A significant increase in *Cyanobacteria* could interrupt the balance of gut microbiota in turbot fed CeO_2_ ENPs-treated clamworms (Fig. 4C), resulting in severe damage to the intestinal mucosa. Additionally, the abundance of *Bacillus*, as the probiotics in aquaculture [39], significantly decreased in turbot fed CeO_2_ ENPs-treated clamworms at the genus level. In this study, low abundances of *Bacillus* and *Lactobacillus* were not beneficial to the intestinal health and nutrient absorption of turbot after feeding CeO_2_ ENPs-treated clamworms. Specifically, increased abundances of *Stenotrophomonas* and *Vibrio* (pathogen), closely associated with fish metabolism, inflammation, and disease [38], were observed in the 100DE group. These findings indicated severe dysregulation of the intestinal microbiota in turbot fed CeO_2_ ENPs-treated clamworms, potentially impairing host functions and health by reducing intestinal nutrient absorption and growth performance while increasing morbidity. Importantly, fish growth is mainly reflected in muscle, such as muscle fibers and collagen, which are tightly related to muscle color, firmness, and elasticity [40]. The growth of muscle fibers and collagen synthesis is related to gut microbiota [41]. Thus, intestinal barrier damage caused by CeO_2_ ENPs may disrupt nutrient digestion and absorption by intestinal microbiota, trigger oxidative stress, impair muscle fiber growth and collagen synthesis, and ultimately reduce growth and flesh quality.

### 3.6 Untargeted metabolomics analysis in muscle and correlation analysis with gut microbiota

**Fig. 5:**
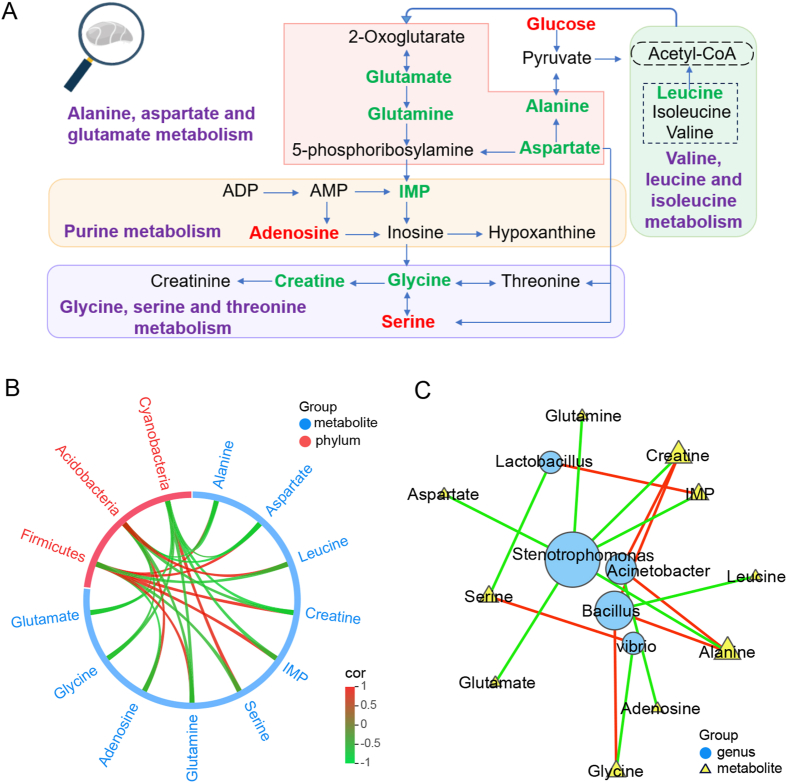
Metabolic pathways of turbot after CeO_2_ ENPs dietary exposure (A) and the correlation between key metabolites and different microorganisms at the phylum level (B) and at the genus level (C), respectively. In panel (A), metabolites in red and green represent significantly higher or lower levels in the muscle of the 100DE group compared to the control group. Metabolites marked in black represent those detected with no significant change. In panel (B, C), Spearman correlation analysis of altered flesh-related metabolites and the induced differences in the intestinal microbiota of turbots after being fed with CeO_2_ ENPs-treated clamworms. The color key denotes Spearman's correlation coefficient, with significant correlations defined by threshold values of |*r*| > 0.7 and *p* ​< ​0.05.

Metabolomics is expected to explain the underlying mechanism of muscle texture changes in fish [14]. We then performed untargeted metabolomics analysis of muscle samples. In this study, principal component analysis (PCA) and orthogonal PLS-DA (OPLS-DA) showed clear separation between the 100DE group and the control, demonstrating significant differences in metabolites between the 100DE and the control group (Fig. S8). Next, changes in the amount of 53 metabolites were identified in the 100DE group versus the control (Table S8). Among these, glucose, inosine monophosphate (IMP), creatine, and several amino acids (alanine, glycine, aspartate, serine, glutamate, and glutamine) showed significant differences in the 100DE group compared to the control group. According to the Kyoto Encyclopedia of Genes and Genomes pathway database, the primary pathways of turbot flesh metabolism affected by CeO_2_ ENPs dietary exposure are sketched and summarized in Fig. 5A.

As shown in Fig. 5A, most metabolites were associated with umami. For the flesh flavor, umami is a very critical component in seafood, which mainly derives from nucleotides and umami amino acids (AAs) [42]. In nucleotides, IMP, AMP, and GMP serve as key components contributing to umami [43]. Among them, IMP can provide umami and sweetness while enhancing diverse flavor profiles in foods even at low concentrations [44]. In free AAs, aspartate and glutamate are categorized as umami AAs [42]. As an intermediate metabolite, IMP is transformed from aspartate and glutamate and is involved in purine metabolism, primarily involving glycine, serine, threonine, and creatine. Glycine, as a sweet AA, also enhances the umami flavor in food [42]. In this study, lower umami AAs (aspartate and glutamate) and sweet AAs (glycine) in the 100DE group resulted in poorer flesh flavor than that in the control group (Fig. S5). Moreover, aspartate and glutamate serve as key excitatory neurotransmitters that enhance fish endurance against fatigue [18]. The downregulation of aspartate and glutamate indicated that the energy state of turbot was weaker after CeO_2_ ENPs dietary exposure, supported by the decreased growth. Furthermore, creatine and betaine influenced WHC, flesh tenderness, and AAs content [42]. Increased creatine content in muscle may delay the formation of lactate, potentially improving WHC [45]. However, lower creatine content in turbot muscle after being fed with CeO_2_ ENPs-treated clamworms may impact muscle performance in this study. Higher lactate content and lower WHC were observed in the 100DE group to support this view. Besides, leucine is a ketogenic branched-chain amino acid, which is indispensable for growth performance, antioxidant capacity, intestinal health, and other physiological functions in fish [46]. Notably, leucine could enhance the nutritional composition of fish muscle, including amino acid composition and collagen content, and improve flesh texture by increasing WHC, muscle shear force, and chewiness [15]. However, a decrease in leucine in the 100DE group verified that turbot fed CeO_2_ ENPs-treated clamworms reduced growth performance and flesh texture. It follows then that glycine, serine, and threonine metabolism, purine metabolism, alanine, valine, leucine, and isoleucine metabolism, and aspartate and glutamate metabolism were identified as key metabolic pathways impacting the flesh quality of turbot fed CeO_2_ ENPs-treated clamworms. Additionally, lipid oxidation plays a critical role in the formation of flesh texture and is positively correlated with ROS [16]. ROS are readily generated in the mitochondria of fish skeletal muscle [14]. For instance, the NAD^+^ level in the mitochondrial matrix of Wuchang-bream muscle downregulates during live transportation, resulting in the accumulation of ROS, leading to oxidative stress-induced alterations in muscle metabolism and texture [14]. In this study, a significant reduction in NAD^+^ was observed in the 100DE group compared with the controls (Table S8), potentially promoting ROS accumulation in muscle tissue. The increased ROS exacerbates lipid oxidation, thereby inhibiting growth performance and deteriorating flesh quality [11,14]. Thus, the deterioration of flesh quality in turbot could be explained by muscle metabolism disorders caused by oxidative stress following exposure to CeO_2_ ENPs.

It is known that the production of animal-derived foods (meat, eggs, and milk) is closely linked to nutrient digestion and absorption mediated by gut microbiota, with active metabolic exchange occurring between microbes and the host. Emerging evidence indicates that gut microbiota affects muscle metabolic pathways to regulate flesh quality via the gut–muscle axis [41]. The relationship between altered flesh-related metabolites and the induced differences in the intestinal microbiota of turbots fed with CeO_2_ ENPs-treated clamworms was further investigated (Fig. 5B and C). Correlation analysis revealed that among phylum-level bacterial communities, *Firmicutes* were positively correlated with creatine (0.81, Spearman correlation coefficient), alanine (0.90), aspartate (0.83), glutamine (0.76) and IMP (0.79), but negatively correlated with serine (−0.71), leucine (−0.83), and adenosine (−0.90). *Acidobacteria* exhibited positive correlations with adenosine (0.71) and leucine (0.81), while showing negative associations with glutamine (−0.71), creatine (−0.76), glycine (−0.79), alanine (−0.90), and aspartate (−0.98). *Cyanobacteria* had a positive correlation with serine (0.76), but significant negative correlations with glutamine (−0.74), glycine (−0.74), aspartate (−0.74), IMP (−0.86), glutamate (−0.88), and creatine (−0.95). Therefore, the high abundance of *Cyanobacteria* posed greater potential risks to metabolites associated with flesh quality. At the genus level, *Lactobacillus* was positively associated with IMP (0.81) but negatively associated with serine (−0.83). *Vibrio* exhibited a positive correlation with serine (0.88) and a negative correlation with glycine (−0.86). *Acinetobacter* was positively correlated with creatine (0.81) and alanine (0.86), while showing a negative correlation with adenosine (0.83). *Bacillus* demonstrated positive correlations with creatine (0.86), alanine (0.83), and glycine (0.83), but a negative correlation with leucine (−0.90). *Stenotrophomonas* was negatively associated with all measured metabolites: glutamate (−0.95), creatine (−0.95), alanine (−0.90), aspartate (−0.83), glutamine (−0.81), and IMP (−0.83). In addition to *Lactobacillus*, *Vibrio*, and *Acinetobacter*, the abundances of *Stenotrophomonas* and *Bacillus* were more strongly correlated with altered flesh-related metabolites. As reported in a previous study, *Stenotrophomonas*, *Lactobacillus*, and *Bacillus* are highly correlated with muscle metabolic pathways (e.g., serine, glucose, and oxidative metabolism) in animals [41]. Thus, microbiota variation caused by CeO_2_ ENP exposure is a key factor affecting muscle metabolites and phenotype in fish via the gut–muscle axis. The growth performance of fish is closely linked to their physiological status [2,5,10]. The decline in growth and nutrient composition of marine fish caused by ENPs may result from increased inflammation and oxidative stress [2,5,47], as well as reduced feeding activities and intestinal digestive capacity [8,17]. Specifically, TiO_2_ ENP exposure altered the abundance of *Lactobacillus* and *Nautella* in the intestine, which was highly correlated with immune factors and amino acid metabolism disorders of the grouper, thereby influencing health status [47]. Then, TiO_2_ ENPs caused damage to the intestinal structure, reducing the muscle nutritional quality of turbot [2]. Overall, this study provides new insights for a better understanding of the trophic transfer of ENPs to assess ecological risk and food safety.

## 4 Conclusions

CeO_2_ ENPs were observed to transfer from benthic invertebrates to fish, with subsequent biotransformation along the marine benthic food chain. Although no biomagnification of CeO_2_ ENPs occurred along this food chain, CeO_2_ ENPs experienced biotransformation from Ce(IV) to Ce(III) in clamworms and turbot feces. Importantly, 3.73% of Ce accumulated in muscle, affecting food safety. During trophic transfer, turbots were severely impaired in growth performance and flesh quality, such as low amino and collagen content, flavor, water-holding capacity, hardness, shear force, and chewiness, showing the deterioration of flesh quality. A decrease in the flesh quality of turbot after CeO_2_ ENPs dietary exposure was potentially related to oxidative stress in muscle and disturbance of intestinal balance, resulting in changes in the physiological metabolism of muscle. The results revealed that purine metabolism, glycine, alanine, aspartate and glutamate metabolism, serine and threonine metabolism and valine, leucine and isoleucine metabolism were the main metabolic pathways impacting turbot flesh quality upon CeO_2_ ENPs dietary exposure. Furthermore, the abundances of *Stenotrophomonas*, *Vibrio*, *Bacillus*, *Lactobacillus*, and *Acinetobacter* were highly correlated with flesh-related metabolite levels (e.g., glutamate, creatine, aspartate, glycine, leucine, and IMP) via the gut–muscle axis. This study highlights the changes in fish flesh texture and flavor as affected by the trophic transfer of ENPs to assess ecological risk and food safety in the environment.

## CRediT authorship contribution statement

**Liyun Yin:** Writing – original draft, Validation, Conceptualization, Writing – review & editing, Visualization, Funding acquisition. **Zhuomiao Liu:** Investigation, Methodology. **Jian Zhao:** Writing – review & editing, Funding acquisition, Supervision. **Shu Chen:** Methodology, Investigation. **Xiaochuan Wang:** Methodology, Investigation. **Zhenyu Wang:** Writing – review & editing.

## Declaration of competing interests

The authors declare that they have no known competing financial interests or personal relationships that could have appeared to influence the work reported in this paper.
