# Supplementary material for: Trophic transfer of CeO2 nanoparticles from clamworm to juvenile turbot and related changes in fish flesh quality
Source: Eco Environ Health. 2025 Aug 5;4(3):100174. doi: 10.1016/j.eehl.2025.100174 (PMC12405684; doi:10.1016/j.eehl.2025.100174)
Supplement: Multimedia component 1 [file mmc1.docx]

**Supporting information**

**Trophic transfer of CeO_2_ nanoparticles from clamworm to juvenile turbot and related changes in fish flesh quality**

Liyun Yin ^a^, Zhuomiao Liu ^b^, Jian Zhao^b,^*, Shu Chen ^a^, Xiaochuan Wang ^a^, Zhenyu Wang^c,^*,

^a^ College of Life Sciences, Hebei Key Laboratory of Animal Physiology, Biochemistry and Molecular Biology, Hebei Collaborative Innovation Center for Eco-Environment, Hebei Normal University, Shijiazhuang 050024, China

^b^ Institute of Coastal Environmental Pollution Control, Laboratory of Marine Environment and Ecology, and Frontiers Science Center for Deep Ocean Multispheres and Earth System, Ocean University of China, Qingdao 266100, China

^c^ Institute of Environmental Processes and Pollution control, and School of Environment and Ecology, Jiangnan University, Wuxi 214122, China

*Corresponding authors

1. mail address: jzhao@ouc.edu.cn (J. Zhao); [wang0628@jiangnan.edu.cn](mailto:wang0628@jiangnan.edu.cn) (Z. Wang)

**Text S1.** Growth performance and body composition analysis for turbot

Growth performance of turbots was calculated as follow [1]:

Weight gain rate (WGR, %) =100× [(final body weight − initial body weight) / initial body weight].

Survival rate (SR, %) =100× (final number of fish / initial number of fish).

Morbidity (%) =100× (number of sick fish / initial number of fish).

Condition factor (CF, %) =100× (fresh body weight, g) / (body length, cm)^3^.

Viscerosomatic index, (VSI, %) =100× (fresh visceral weight / fresh body weight).

Hepatosomatic index, (HSI, %) =100× (fresh liver weight/fresh body weight).

According to the Association of Official Analytical Chemists (AOAC, 2010) [2], the proximate composition of fish body was analyzed. For moisture, the whole fish were dried to a constant weight at 105 °C in a drying oven. Subsequently, the dry samples of whole body were grounded, and the crude protein content (N × 6.25) and crude lipid content of whole body were determined using Kjeldahl and the chloroform-methanol method, respectively. Ash content was determined by combusting samples in muffle furnace at 550 °C for 6 h.

**Text S2.** Gut microbiota analysis of turbot

Bacterial DNA of the intestinal samples was extracted using an Omega Mag-bind soil DNA kit (Omega Bio-Tek, U.S.A.), and DNA concentrations were assessed with a NanoDrop spectrophotometer (Thermo Scientific, U.S.A.). The integrity of the DNA was analyzed by electrophoresis. Specific primers (5’-CCTACGGGNGGCWGCAG-3’ and 806R: 5’-GGACTACHVGGGTATCTAAT-3’) were used to amplify the V3–V4 region of the DNA sample. The sequencing library was sequenced on the Illumina NovaSeq Instrument. Sequencing results had barcodes and primers removed using in-house scripts. Data filtering of high-quality sequences strictly followed the QIIME2 quality control process. Operational taxonomic units (OTUs) were determined based on a sequence identity of 97%. Alpha diversity was calculated using the Chao1 index, Simpson index, Shannon index, etc. Beta diversity was determined through principal coordinate analysis (PCoA) to compare the differences in microbial communities between control and 100DE samples.

**Table S1.** The surface-adsorbed Ce contents (μg/g, dry weight) of clamworms. The dead clamworms were exposed to CeO_2_ ENPs (10, 50, 100 mg/L) for 24 hours, then rinsed four times with deionized water prior to digestion and Ce determination.

| CeO_2_ ENPs  (mg/L) | Ce content in dead clamworms  (μg/ g, dry weight) |
| --- | --- |
| 10 | 4.53 ± 0.66 ^N.S.^ |
| 50 | 5.17±0.96 ^N.S.^ |
| 100 | 5.35±0.39 ^N.S.^ |

Note: N.S. = not statistically significant (*n* = 4, *p* > 0.05).


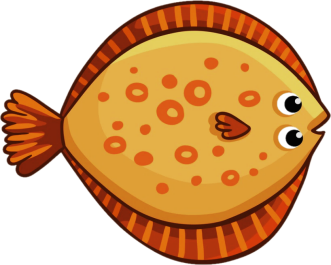


**Fig. S1** The dorsal muscle from the body in the same position was collected to flesh quality analyses.


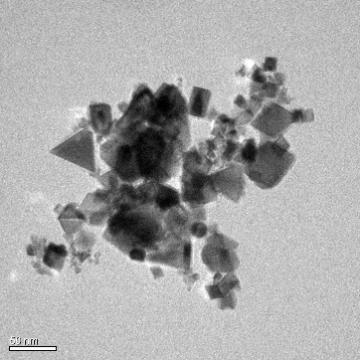

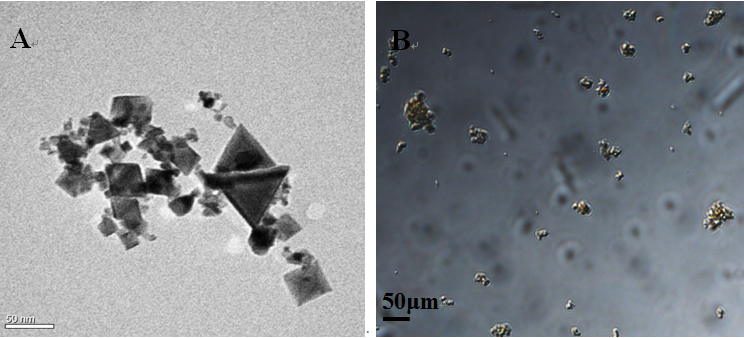


A

**Fig. S2** Transmission electron microscopy image of CeO_2_ ENPs (A) and light microcopy image of CeO_2_ ENPs in seawater (B).

**Table S2.** Selected physicochemical properties of CeO_2_ ENPs

| NPs | Particle size  (nm) | Surface area  (m^2^/g) | Hydrodynamic diameter  (nm) | | Zeta potential  (mV) | | Ionic Ce^c^  (mg/L) |
| --- | --- | --- | --- | --- | --- | --- | --- |
|  |  |  | DI water | Seawater | DI Water | Seawater | Seawater |
| CeO_2_ | <25^a^ | 41.5 | 132 ± 1 | 1647 ± 16 | 23.2 ± 0.3 | ----^b^ | Not detected |

^a^ Particle size of individual CeO_2_ ENPs was obtained from TEM imaging (Figure S1A);

^b^ Zeta potential of CeO_2_ ENPs was not available because of poor dispersion in seawater;

^c^ The ionic Ce was released from CeO_2_ ENPs (100 mg/L) after 24 hours dissolution.


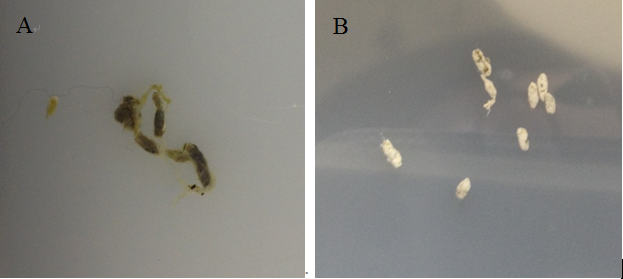


**Fig. S3** Feces of clamworms collected from un-exposed (A) and CeO_2_ ENPs-exposed (B) treatments. For CeO_2_ ENPs-exposed treatment, clamworms were treated with CeO_2_ ENPs (100 mg/L) for 1 day. After washing, pre-clamworms were depuration in clean seawater for 24 hours. The excreted feces from the clamworms were then collected.

**Table S3.** Bioconcentration Factors (BCFs) of Ce in clamworms after exposure to CeO_2_ ENPs (0, 10, 50 and 100 mg/L) for 24 h.

| CeO_2_ ENPs  (mg/L) | Bioconcentration Factors (BCFs) |
| --- | --- |
| 0 | ---- |
| 10 | 36.22 ±5.55^b^ |
| 50 | 57.02±1.06^a^ |
| 100 | 34.30 ±1.94^b^ |

Note: different letters indicate significant difference among different treatments (*n* = 3, *p* < 0.05).

**Table S4.** Influence of CeO_2_ ENPs waterborne exposure on the nutritional status of clamworms (%)

| Concentrations | 0 mg/L | 10 mg/L | 50 mg/L | 100 mg/L |
| --- | --- | --- | --- | --- |
| Survival rate | 100 | 100 | 100 | 100 |
| Moisture | 80.23 ± 3.76^N.S.^ | 80.17 ± 4.15^N.S^ | 80.27 ± 3.83^N.S^ | 80.14 ± 4.09^N.S^ |
| Crude protein | 52.03 ± 2.27^N.S.^ | 51.53 ± 3.05^N.S.^ | 51.85 ± 2.07^N.S.^ | 52.07 ± 2.53^N.S.^ |
| Crude lipid | 9.68 ± 0.51^N.S.^ | 9.81 ± 0.43^N.S^ | 9.54 ± 0.62^N.S^ | 9.73 ± 0.49^N.S^ |

Note: N.S. = not statistically significant (*n* = 4, *p* > 0.05). Crude protein and crude lipid are dry weight (%).

**Fig. S4** Ce content in (A) liver, (B) stomach, (C) intestines, (D) gill, (E) muscle, (F) skin, (G) blood, (ng/ml) of juvenile turbot after CeO_2_ ENPs dietary (10DE, 50DE, and 100DE) exposure for 20 days. (H) Ce content in feces for 27 days. For a given organ/tissue, different letters represent significant difference among different treatments (*n* = 3, *p* < 0.05).

**Table S5.** Influence of CeO_2_ ENPs dietary exposure on growth performance and survival of juvenile turbot for 27 days.

| Concentrations | Control | 10DE | 50DE | 100DE |
| --- | --- | --- | --- | --- |
| IBW (g/fish) | 42.8±0.9 | 42.1±0.5 | 42.1±0.9 | 42.7±0.6 |
| FBW(g/fish) | 45.4±1.0 | 44.5±0.5 | 44.4±0.9 | 44.96±0.69 |
| WGR (%) | 6.00±0.23 ^a^ | 5.81±0.12 ^a^ | 5.41±0.20 ^b^ | 5.23±0.27 ^b^ |
| SR (%) | 100% | 100% | 100% | 100% |
| Morbidity (%) | 5.56% ^a^ | 16.67% ^b^ | 27.78% ^c^ | 36.11% ^d^ |
| CF (%) | 3.16±0.16 | 3.17±0.13 | 3.22±0.22 | 3.12±0.16 |
| VSI (%) | 4.36±0.14 | 4.36±0.10 | 4.35±0.20 | 4.34±0.16 |
| HSI (%) | 1.36±0.08 a | 1.41±0.10 a | 1.36 ±0.13 a | 1.57±0.14 b |

Note: IBW, initial body weight; FBW, final body weight. Morbidity refers to the phenomenon of fish with rotten tails. For a given parameter, different letters indicate significant difference among different concentrations of CeO_2_ ENPs dietary exposures (*n* = 3, *p* < 0.05).

**Table S6.** Influence of CeO_2_ ENPs dietary exposure on body composition of juvenile turbot (%)

| Concentrations | Control | 10DE | 50DE | 100DE |
| --- | --- | --- | --- | --- |
| Moisture | 78.3±1.1 | 78.1±1.1 | 78.2±1.6 | 78.5±1.6 |
| Crude protein | 14.1±0.19 ^a^ | 13.97±0.22 ^a^ | 13.78±0.20 ^b^ | 13.64±0.23 ^b^ |
| Crude lipid | 4.16±0.12 ^a^ | 4.25±0.17 ^a^ | 4.51±0.13 ^b^ | 4.61±0.13 ^b^ |
| Ash | 3.24±0.13 | 3.43±0.22 | 3.39±0.19 | 3.39±0.29 |

Note: For a given parameter, different letters indicate significant difference among different concentrations of CeO_2_ ENPs dietary exposures (*n* = 3, *p* <0.05).

**Table S7.** Influence of CeO_2_ ENPs dietary exposure on muscle acid compositions (g/100g, wet weight) of juvenile turbot

| Concentrations | Control | 10DE | 50DE | 100DE |
| --- | --- | --- | --- | --- |
| TEAAs | 29.99 ± 0.91^a^ | 29.66 ± 0.36^ab^ | 29.44 ± 0.61^ab^ | 28.82 ± 0.31^b^ |
| TNEAAs | 41.97 ± 0.46^a^ | 41.57 ± 0.63^ab^ | 40.55 ± 0.69^b^ | 40.46 ± 0.90^b^ |
| TAAs | 72.06 ± 0.85^a^ | 71.26 ± 1.12^ab^ | 69.99 ± 1.20^b^ | 69.29 ± 1.18^b^ |
| DAAs | 34.48 ± 0.75^a^ | 34.29 ± 0.38^ab^ | 33.88 ± 0.40^ab^ | 33.41 ± 0.43^b^ |
| TEAAs/TAAs (%) | 41.88 ± 2.39^a^ | 41.75 ± 2.49^a^ | 41.72 ± 2.32^a^ | 41.24 ± 1.36^a^ |
| TEAAs/TNEAAs (%) | 72.76 ± 0.75^a^ | 71.69 ± 1.33^ab^ | 71.42 ± 1.34^ab^ | 70.27 ± 1.17^b^ |

Note: EAAs, essential amino acids; NEAAs, total non-essential amino acids; TEAAs, total essential amino acids; TNEAAs, total non-essential amino acids; TAAs, total amino acids; DAAs, delicious amino acids; Different letters indicate significant difference (*n* = 3, *p* < 0.05).

**Fig. S5** Amino acid content (g/100g, wet weight) of juvenile turbot after CeO_2_ ENPs dietary exposure for 27 days. different letters represent significant difference among different treatments (*n* = 3, *p* <0.05).


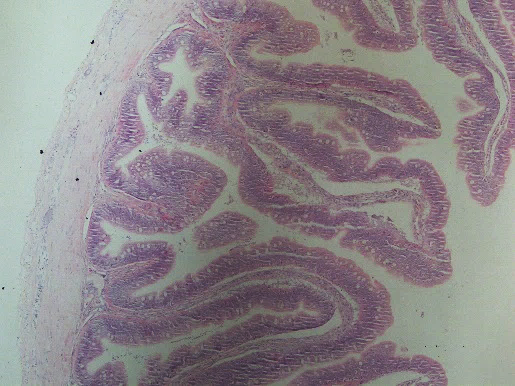

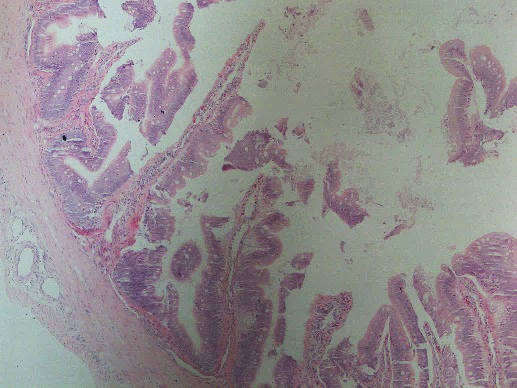


B

A

**Fig. S6** Pathological changes in intestine of juvenile turbot after CeO_2_ ENPs dietary exposure (100DE) for 27 days. (A) Control, (B) 100DE.


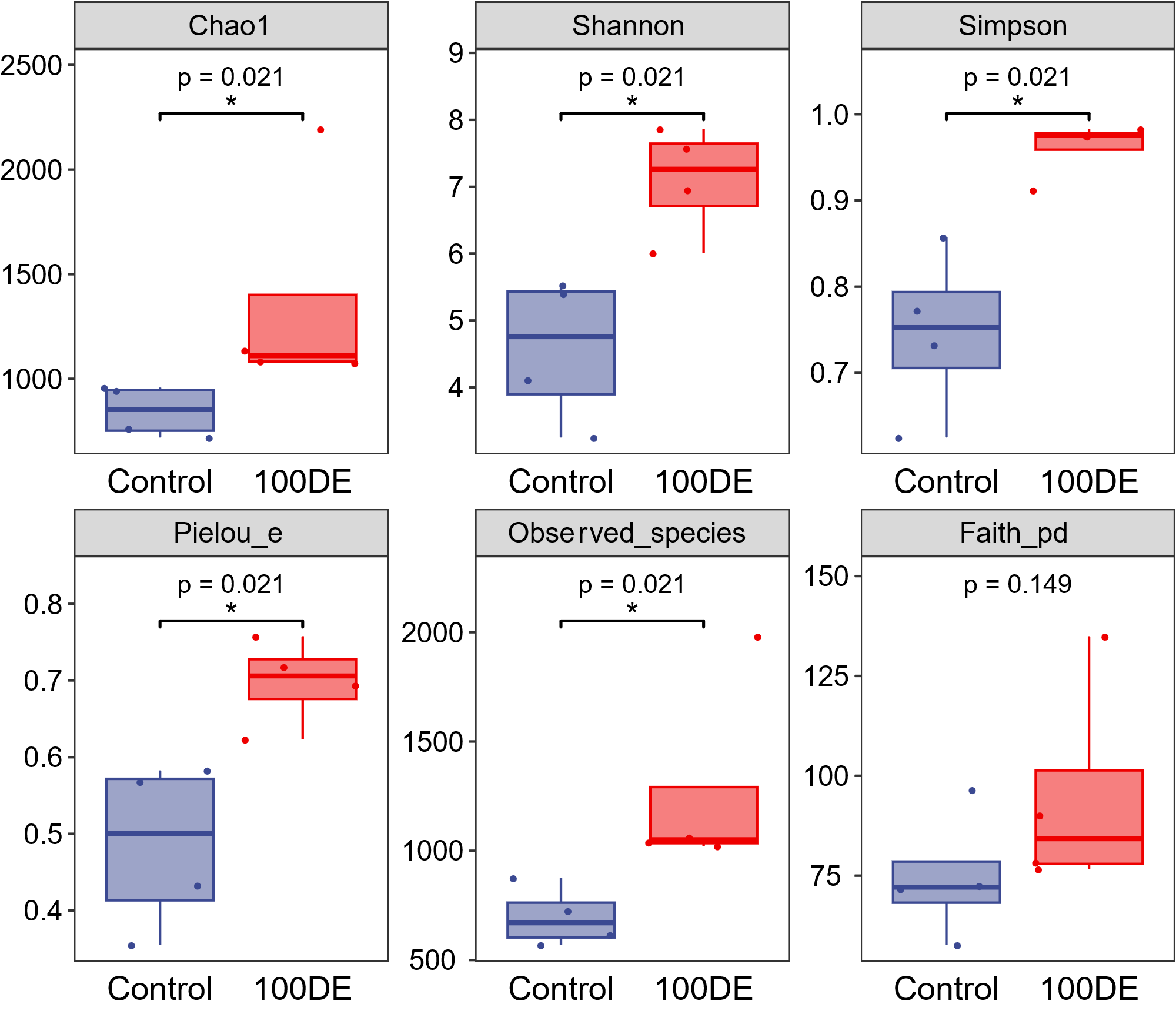


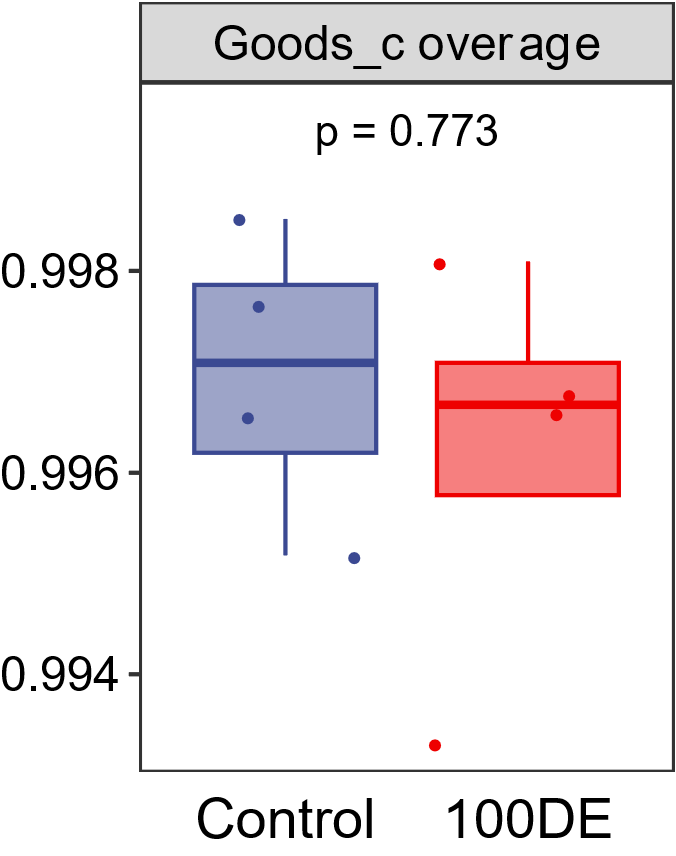

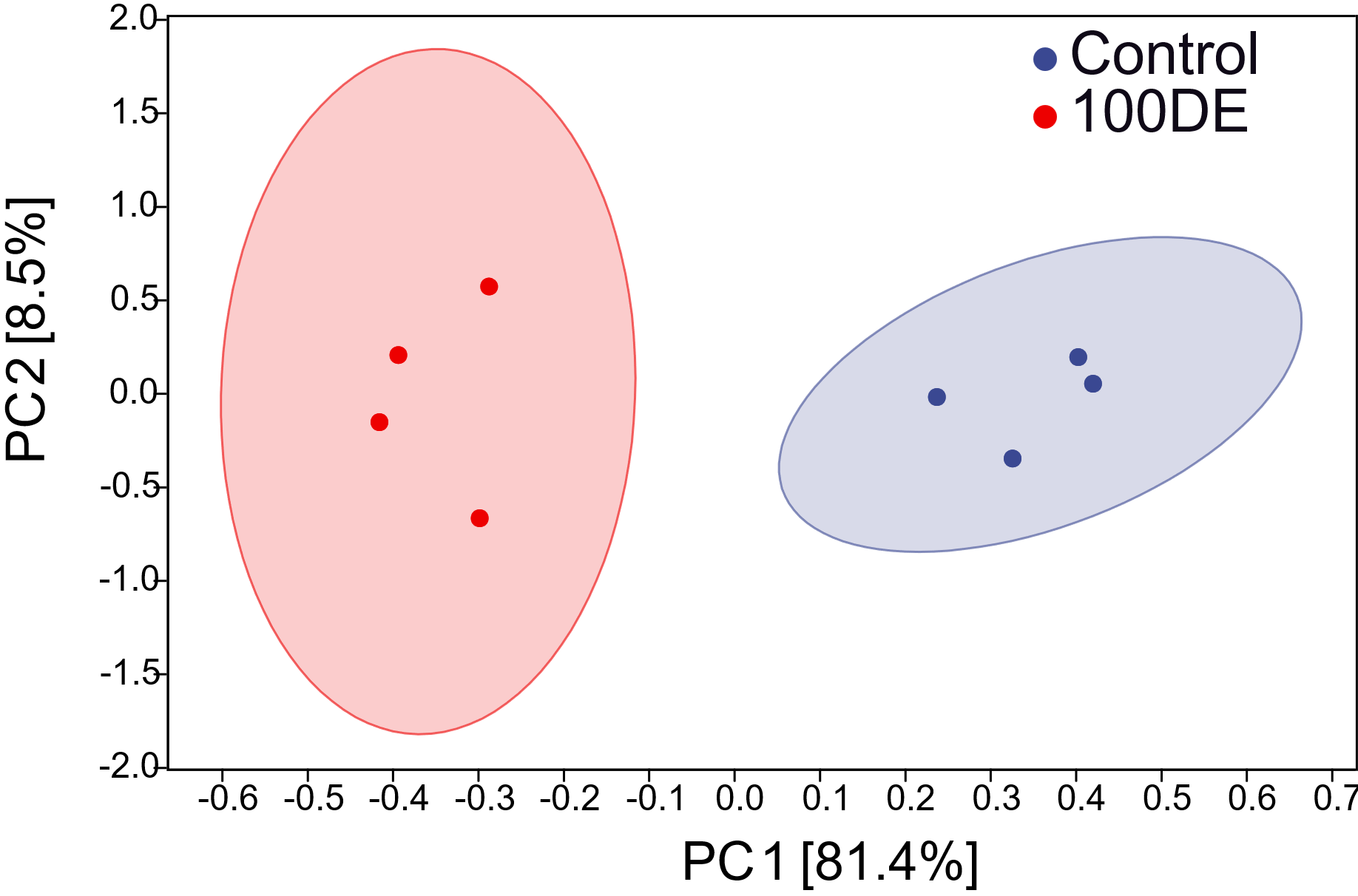


**Fig. S7** The diversity and community composition of intestinal microbes in juvenile turbot after CeO_2_ ENPs dietary exposure for 27 days between control and 100DE groups.

A PCA


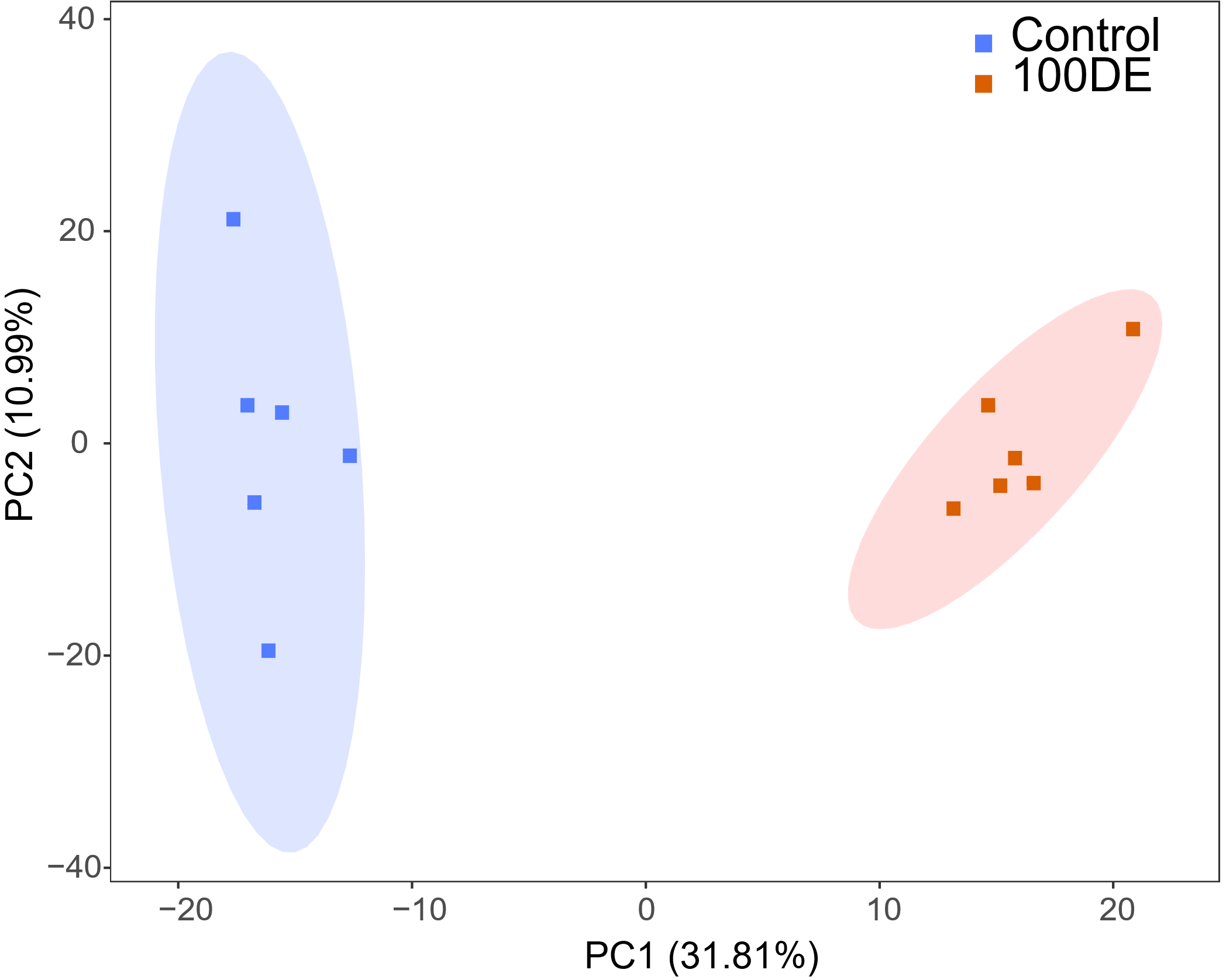

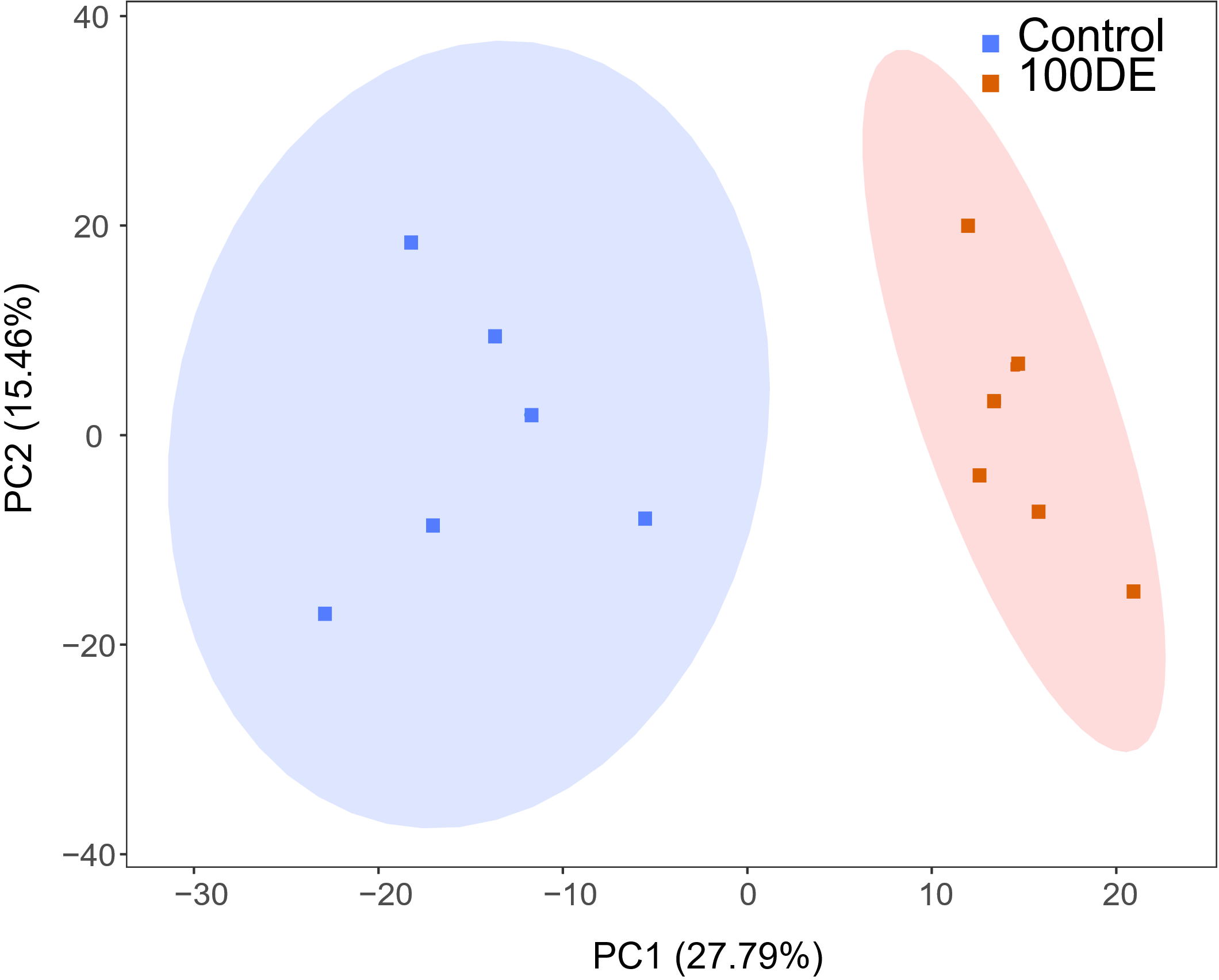


Positive Negative

B OPLS-DA


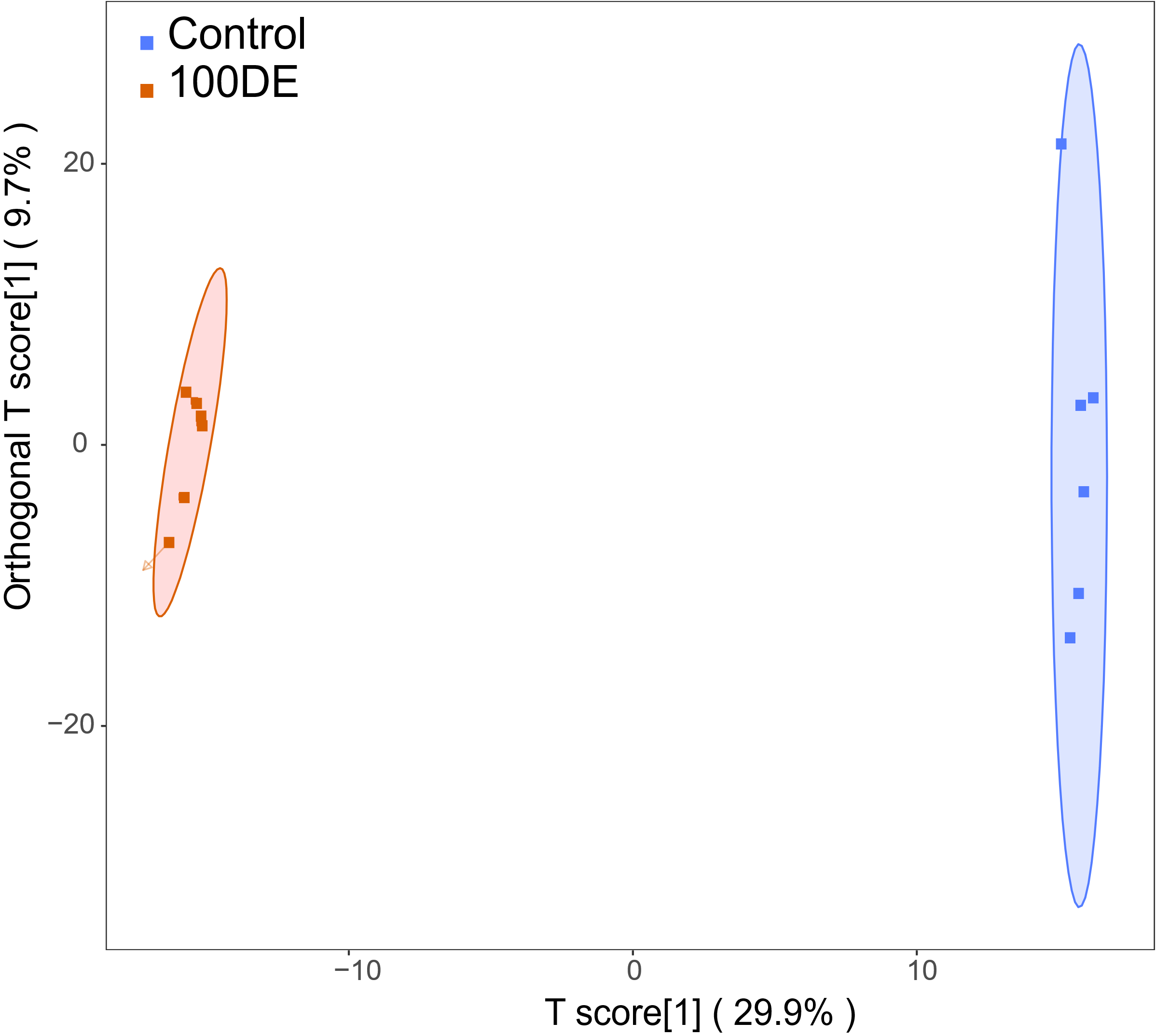

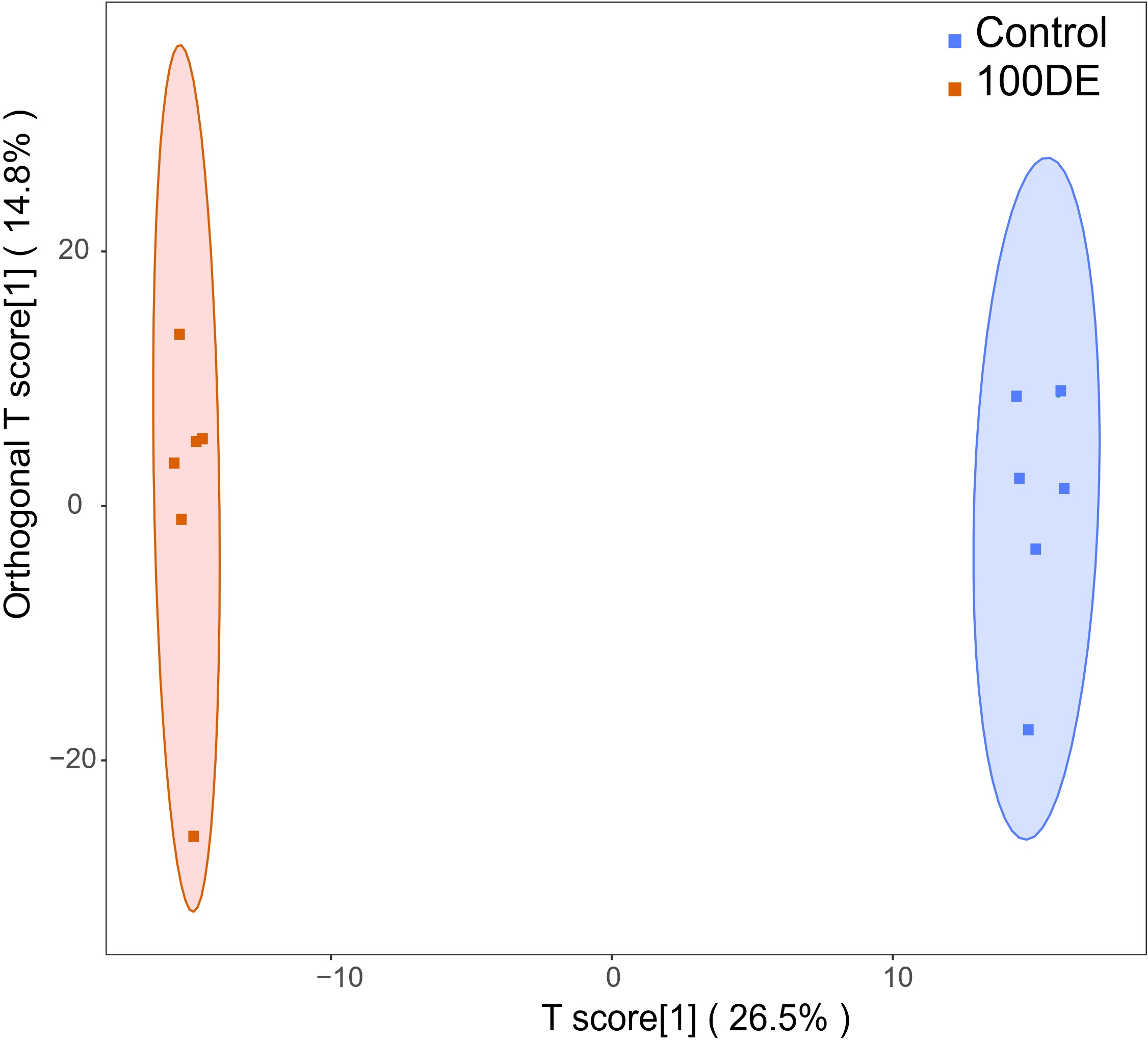


Positive Negative

**Fig. S8** The PCA (A) and OPLS-DA (B) score plots of juvenile turbot muscle after CeO_2_ ENPs dietary exposure for 27 days between control and100DE groups in positive ion mode (left) and negative ion mode (right). In OPLS-DA, Positive, R^2^X=0.396, R^2^Y=0.999, Q^2^=0.929, *p* <0.005. Negative, R^2^X=0.413, R^2^Y=0.999, Q^2^=0.905, *p* < 0.005.

**Table S8.** The different metabolites of juvenile turbot muscle after CeO_2_ ENPs dietary exposure for 27 days between control and100DE groups

| Compounds | VIP | P-value | Fold_  Change | Log_2_FC | Type |
| --- | --- | --- | --- | --- | --- |
| Indole-3-acetonitrile | 1.7996 | 0.0000 | 15.6017 | 3.9636 | up |
| Glycocholic acid | 1.2031 | 0.0339 | 3.7267 | 1.8979 | up |
| (S)-O-Desmethyl Naproxen | 1.6719 | 0.0007 | 22.1216 | 4.4674 | up |
| D-glucose | 1.7395 | 0.0000 | 5.5418 | 2.4704 | up |
| Purine | 1.2808 | 0.0009 | 4.1121 | 2.0399 | up |
| UDP-D-galactose | 1.0720 | 0.0097 | 2.7012 | 1.4336 | up |
| [[(2R,3S,4R,5R)-5-(6-aminopurin-9-yl)-3,4-dihydroxyoxolan-2-yl]methoxy-hydroxyphosphoryl]5-[(4S)-2-oxo-1,3,3a,4,6,6a-hexahydrothieno[3,4-d]imidazol-4-yl]pentanoate | 1.2209 | 0.0030 | 3.0761 | 1.6211 | up |
| Se-Adenosylselenohomocysteine | 1.2865 | 0.0005 | 2.6569 | 1.4098 | up |
| L-serine | 1.5929 | 0.0388 | 2.7746 | 1.4723 | up |
| (2R,6S)-2,6-Diaminoheptanedioic acid | 1.5887 | 0.0412 | 3.2905 | 1.7183 | up |
| Adenosine | 1.3770 | 0.0223 | 2.5962 | 1.3764 | up |
| Guanosine 5'-Diphosphate Sodium Salt | 1.8389 | 0.0000 | 2.2297 | 1.1569 | up |
| Xanthosine 5'-(trihydrogen diphosphate) | 1.5308 | 0.0004 | 2.3657 | 1.2423 | up |
| Taurochenodeoxycholate-7-sulfate | 1.8043 | 0.0038 | 4.7099 | 2.2357 | up |
| 3-Phosphonoalanine | 1.4111 | 0.0000 | 5.2086 | 2.3890 | up |
| Nicotinic Acid | 1.5734 | 0.0079 | 3.6965 | 1.8862 | up |
| Leukotriene F4 | 1.3274 | 0.0001 | 5.2038 | 2.3796 | up |
| 6'-Malonyltrifolirhizin | 1.7629 | 0.0000 | 12.6889 | 3.6655 | up |
| 1alpha,23(S),25-trihydroxyvitamin D3 | 1.2387 | 0.0199 | 4.6272 | 2.2102 | up |
| N-Acetyl-D-glucosamine 6-phosphate | 1.4987 | 0.0092 | 2.0863 | 1.0609 | up |
| alpha-Ergocryptine | 1.7774 | 0.0024 | 3.5610 | 1.8323 | up |
| Mesoxalic acid | 1.8836 | 0.0000 | 2.2451 | 1.1668 | up |
| 3-Oxotetradecanoic acid | 1.8243 | 0.0019 | 6.1473 | 2.6199 | up |
| 1,2-Di-(9Z-tetradecenoyl)-sn-glycero-3-phosphocholine | 1.0033 | 0.0054 | 23.8431 | 4.5755 | up |
| 1-Stearoyl-2-hydroxy-sn-glycero-3-phosphocholine | 1.0542 | 0.0118 | 2.6028 | 1.3801 | up |
| 1,2-Ditetradecanoyl-sn-glycero-3-phosphocholine | 1.3238 | 0.0047 | 28.0042 | 4.8076 | up |
| S-(5'-Adenosyl)-L-homocysteine | 1.5991 | 0.0001 | 0.1209 | -3.0477 | down |
| Vitamin K | 1.7142 | 0.0088 | 0.0161 | -5.9599 | down |
| Xanthurenic Acid | 1.4083 | 0.0034 | 0.4042 | -1.3068 | down |
| UDP-xylose | 1.6961 | 0.0010 | 0.0485 | -4.3660 | down |
| 4-CDP-2-C-methyl-D-erythritol | 1.6988 | 0.0016 | 0.0875 | -3.5149 | down |
| 2,4-Dioxotetrahydropyrimidine D-ribonucleotide | 1.4238 | 0.0064 | 0.2175 | -2.2012 | down |
| Leucine | 1.4790 | 0.0022 | 4.6514 | -2.2177 | down |
| 9,10-DiHOME | 1.4113 | 0.0228 | 0.4876 | -1.0363 | down |
| 9(S),12(S),13(S)-TriHOME | 1.4455 | 0.0370 | 0.3227 | -1.6319 | down |
| L-Glutamate | 1.8465 | 0.0080 | 0.0916 | -3.4485 | down |
| N-Acetyl-O-demethylpuromycin-5'-phosphate | 1.8712 | 0.0012 | 0.1530 | -2.7089 | down |
| Adenosine 5'-diphosphoribose | 1.1867 | 0.0327 | 0.4517 | -1.1467 | down |
| Tauroursodeoxycholic acid | 1.8173 | 0.0003 | 0.0549 | -4.1859 | down |
| Creatine | 1.6631 | 0.0028 | 0.3503 | -1.5134 | down |
| Alanine | 1.2804 | 0.0200 | 3.0720 | 1.6192 | down |
| Glycine | 1.1855 | 0.0340 | 0.3550 | -1.4940 | down |
| Aspartate | 1.6364 | 0.0209 | 3.5996 | -1.8479 | down |
| Glutamine | 1.5511 | 0.0068 | 0.4761 | -1.071 | down |
| IMP | 1.5453 | 0.0167 | 0.1332 | -2.9080 | down |
| NAD+ | 1.1912 | 0.0141 | 0.3810 | -1.3920 | down |
| Glycolithocholic acid | 1.3672 | 0.0278 | 0.0221 | -5.5010 | down |
| Nicotinate mononucleotide | 1.6541 | 0.0056 | 0.0471 | -4.4092 | down |
| Spirolide B | 1.7263 | 0.0021 | 0.0151 | -6.0493 | down |
| Amastatin | 1.4670 | 0.0043 | 0.3259 | -1.6173 | down |
| 1,2-dioleoyl-sn-Glycero-3-Phosphate | 1.7658 | 0.0023 | 0.0778 | -3.6840 | down |
| 1-Lignoceroyl-2-hydroxy-sn-glycero-3-phosphocholine | 1.7143 | 0.0001 | 0.0500 | -4.3211 | down |
| 1,2-Dipalmitoleoyl-sn-glycero-3-phosphocholine | 1.1244 | 0.0399 | 0.4298 | -1.2181 | down |

**References**

1. L. Yin, B. Chen, B. Xia, X. Shi, K. Qu, Polystyrene microplastics alter the behavior, energy reserve and nutritional composition of marine jacopever (*Sebastes schlegelii*). J. Hazard. Mater. 360 (2018) 97-105.

2. AOAC (Association of Official Analytical Chemists), Official Methods of Analysis, 18^th^ ed. Association of Official Analytical Chemists, Arlington, VA, USA (2010).
